# Decrease in social cohesion in a colonial seabird under a perturbation regime

**DOI:** 10.1038/s41598-020-75259-3

**Published:** 2020-10-30

**Authors:** M. Genovart, O. Gimenez, A. Bertolero, R. Choquet, D. Oro, R. Pradel

**Affiliations:** 1 CEAB (CSIC), Accés Cala Sant Francesc 14, 17300 Blanes, Spain; 2 IMEDEA (CSIC-UIB), Miquel Marquès 21, 07190 Esporles, Spain; 3 CEFE, CNRS, Univ. Montpellier, Univ. Paul Valéry Montpellier 3, EPHE, IRD, 34293 Montpellier, France; 4 Associació Ornitològica Picampall de les Terres de l’Ebre, 43580 Deltebre, Spain

**Keywords:** Conservation biology, Population dynamics

## Abstract

Social interactions, through influence on behavioural processes, can play an important role in populations’ resilience (i.e. ability to cope with perturbations). However little is known about the effects of perturbations on the strength of social cohesion in wild populations. Long-term associations between individuals may reflect the existence of social cohesion for seizing the evolutionary advantages of social living. We explore the existence of social cohesion and its dynamics under perturbations by analysing long-term social associations, in a colonial seabird, the Audouin’s gull *Larus audouinii,* living in a site experiencing a shift to a perturbed regime. Our goals were namely (1) to uncover the occurrence of long-term social ties (i.e. associations) between individuals and (2) to examine whether the perturbation regime affected this form of social cohesion. We analysed a dataset of more than 3500 individuals from 25 years of monitoring by means of contingency tables and within the Social Network Analysis framework. We showed that associations between individuals are not only due to philopatry or random gregariousness but that there are social ties between individuals over the years. Furthermore, social cohesion decreased under the perturbation regime. We sustain that perturbations may lead not only to changes in individuals’ behaviour and fitness but also to a change in populations’ social cohesion. The consequences of decreasing social cohesion are still not well understood, but they can be critical for the population dynamics of social species.

## Introduction

Ecosystems are subject to perturbations, both natural and human-induced, affecting individuals, populations, and communities^1^. When they are strong or are maintained through time, these perturbations may cause a shift between stable states at the level of both individual and population and even lead to population collapses and extinctions^2,3^. Understanding how individuals and populations will respond to these perturbations is critical both from a ‘pure’ ecological standpoint and also from an applied point of view to mitigating the effects of global change^4–6^. Population dynamics may be directly affected by these perturbations through a decrease in demographic parameters such as survival or fecundity, or by a change, immediate or delayed, on individual behaviour, such as an increase in dispersal^7^. Resilience is a key concept used by various scientific disciplines, describing systems' abilities to cope with perturbations, thus, to recover the original stable state after the perturbation^8,9^. We define here population resilience as the maximal pulse perturbation a population can tolerate or absorb without going extinct^1,2^. In social animals, social behavioural processes, such as information sharing and decision-making, add another dimension to understanding the resilience of populations facing perturbations. For instance, the amount of social information can be enhanced not only by positive density-dependence but also by social cohesion^10–13^, defined here as the strength of associative ties between group members. Social cohesion favors the exchange of private information and consequently reduces uncertainty in resource acquisition (e.g. shelter against predators, food, mates) or in decision-making in the face of disturbances, such as dispersal to non-perturbed or less perturbed sites^7,14–16^. Thus, the structure of a group may affect social interactions, information transfer, and collective decisions^17^. Recent studies also show that spatial cohesion may be risk-sensitive and that responses may differ depending on the risk and the social group^18^. Some previous studies have considered how the loss of individuals might affect social cohesion^19–23^. However, more studies are needed to better understand the effects of environmental perturbations on the cohesion of social groups in empirical studies of social animals^24^.

The analysis of social relationships in animal populations may include a range of social dynamics, from simple and ephemeral contacts to permanent and strong bonds between individuals^13,25–28^. Coloniality is a life-history strategy where individuals show a social link among conspecifics by breeding in large and dense groups^29,30^. However, many colonial species are philopatric, thus annual association in breeding places between individuals may not necessarily reflect individual social ties but a shared tendency to breed in the same birthplace (i.e. natal philopatry) or breeding place (i.e. breeding philopatry). Additionally, the decision to join or leave a particular group may be based on general preferences for being with others (gregariousness) or preferences for particular individuals within the subgroup^28^ (i.e. social ties). Thus the challenge lies in disentangling whether an annual association between individuals is only due to philopatry (natal or breeding), to random gregariousness, or also due to the existence of social ties within groups of individuals over time^31^. The existence of social ties between neighbouring pairs in breeding colonies are rarely considered in behavioural and ecological studies^32^ and, if true, such associations may suggest the evolution of social cohesion for exploiting the evolutionary advantages of social living (including social information sharing) for individual fitness prospects. Many colonial seabirds evolved in very variable environments, with very patchy and unpredictable resources (both food and safe breeding habitats), which means that they are forced to make decisions constantly, and social coping and social information sharing may be more relevant than previously thought^4,24^.

Social network theory, which originated in sociology to study human relationships and social organization^11,33,34^ now provides both a conceptual framework and the analytical tools to explore social cohesion and social processes in animal populations^20,35–38^. Network theory is now being simultaneously developed in a number of fields, including statistical physics, sociology, molecular biology, and computer science. As a result, the field is changing at a rapid pace. While not all developments can or should be applied toward the study of animal societies^39^, this rush of novel ideas from other disciplines is enriching behavioural ecology^40^.

To assess the existence of social cohesion and its dynamics under perturbations, we studied an ecological system involving a colonial, social vertebrate (the Audouin’s gull *Larus audouinii*) living in a site experiencing a shift to a perturbed regime. Interestingly from a social point of view, the species breeds aggregated in spatially discrete patches within large colonies. Each breeding season, some patches go extinct and some are colonized^41,42^, with a patch occupation expectancy over time of 1.5 years^24^, forcing individuals to breed in patches different from the ones they were born in or they bred in the previous year. These colonization-extinction processes dismiss the possibility of aggregation only due to natal or breeding philopatry. Additionally, all patches have very similar habitat features, dismissing also the possibility of aggregation due to similar habitat choices between individuals, an important issue when analysing social aggregation in many study systems. Thus, our particular study system may allow us to disentangle whether annual breeding aggregation among individuals is an annual random association, or it rather results from social cohesion among individuals over the years (i.e. there are social ties between individuals).

**Figure 1:**
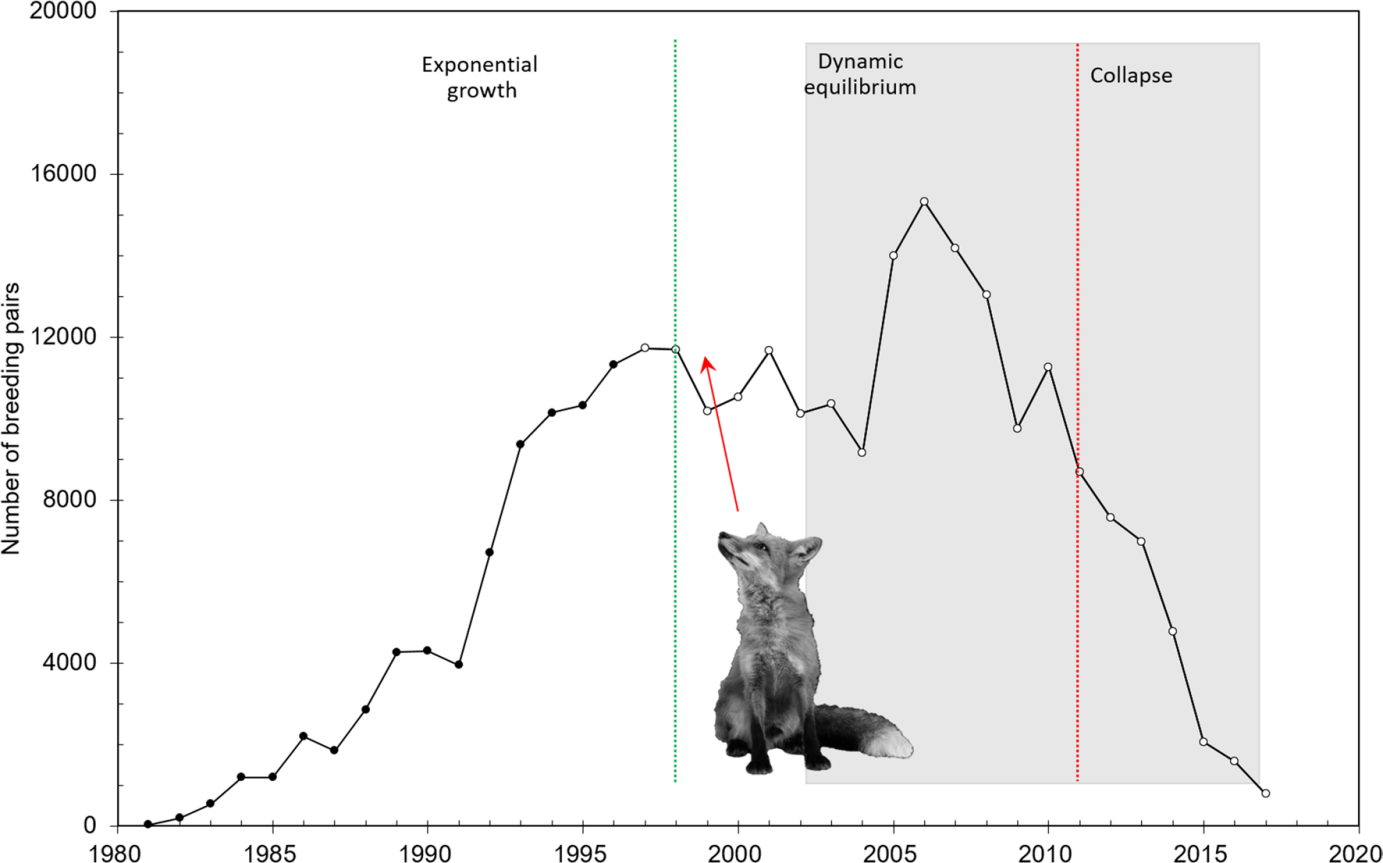
Number of breeding pairs in the Punta de la Banya colony from colonization in 1981 to 2017. The observed phases in the population dynamics (initial growth, dynamic equilibrium and collapse) are separated by dashed lines^44^. Red arrow indicates the arrival of predators (mainly foxes) to the colony. The shadow area shows the temporal window for data analysis for comparing the periods before and over the collapse (separated by a red dashed line).

An extensive long-term individual monitoring program has been carried out since 1988 at the Ebro Delta, including the main breeding site for the species during the last two decades, the Punta de la Banya (40° 34′ 48.0" N 0° 41′ 24.0" E)^43^. At this breeding site, population dynamics have undergone different phases: an initial growing phase after site colonization, a stable phase of dynamic equilibrium, and a final transition phase to population collapse^24,44^ (Fig. 1). This collapse was due to the arrival of terrestrial predators, which led this colony to hold from 70% to only 3% of the total world population in only a decade (32% mean annual decline)^41,43^ (Fig. 1).

The perturbation regime caused changes in the spatial distribution of patches at the site, changes in age structure, decrease in fecundity, and a progressive increase of dispersal to other sites^7,41^. The response of this population to predators has not been immediate probably due to strong philopatry, high site-suitability inertia, and social behavioural processes, such as conspecific attraction^24^. This raise in dispersal was probably caused by social processes, as social copying^24^, however it remains to assess how social cohesion among individuals, if it occurred, was affected by dispersal processes. One possibility is that dispersal would break social cohesion by individual heterogeneity in the willingness to disperse^45^. In contrast, social cohesion can be maintained over time when dispersal between breeding patches occurs collectively at the scale of social groups to the same sites^31,46^. Taking advantage of the long-term monitoring of this species, the knowledge of its population dynamics, and the use of tools recently developed in the Social Network Analysis (SNA) framework, we specifically addressed the following questions: (1) are there any long-term social ties between individuals breeding in the same patch?; and (2) have perturbations, in this case a perturbation regime, affected social cohesion? We finally discuss the role and consequences of social cohesion in population dynamics and resilience in social species.

## Results

We analysed a total of 1,610,922 dyadic interactions during the first period (2002–2011) and 368,142 during the second period (2012–2017) (Supplementary Table S1).

### Occurrence of social ties that persist over time

When assessing the social ties with the contingency table approach during the period of stability, the assumption that breeding aggregations in Audouin’s gull were at random was rejected, and those individuals that bred in the same patch during the sub-period 2002–2006 had a higher probability of breeding together during the sub-period 2007–2011 (${\chi }_{1}^{2}$= 64.685, *P* < 0.0001) (Supplementary Table S1). When randomly reducing the sample size of the data set, results were still statistically significant in more than 95% of the cases (1000 randomizations).

**Figure 2:**
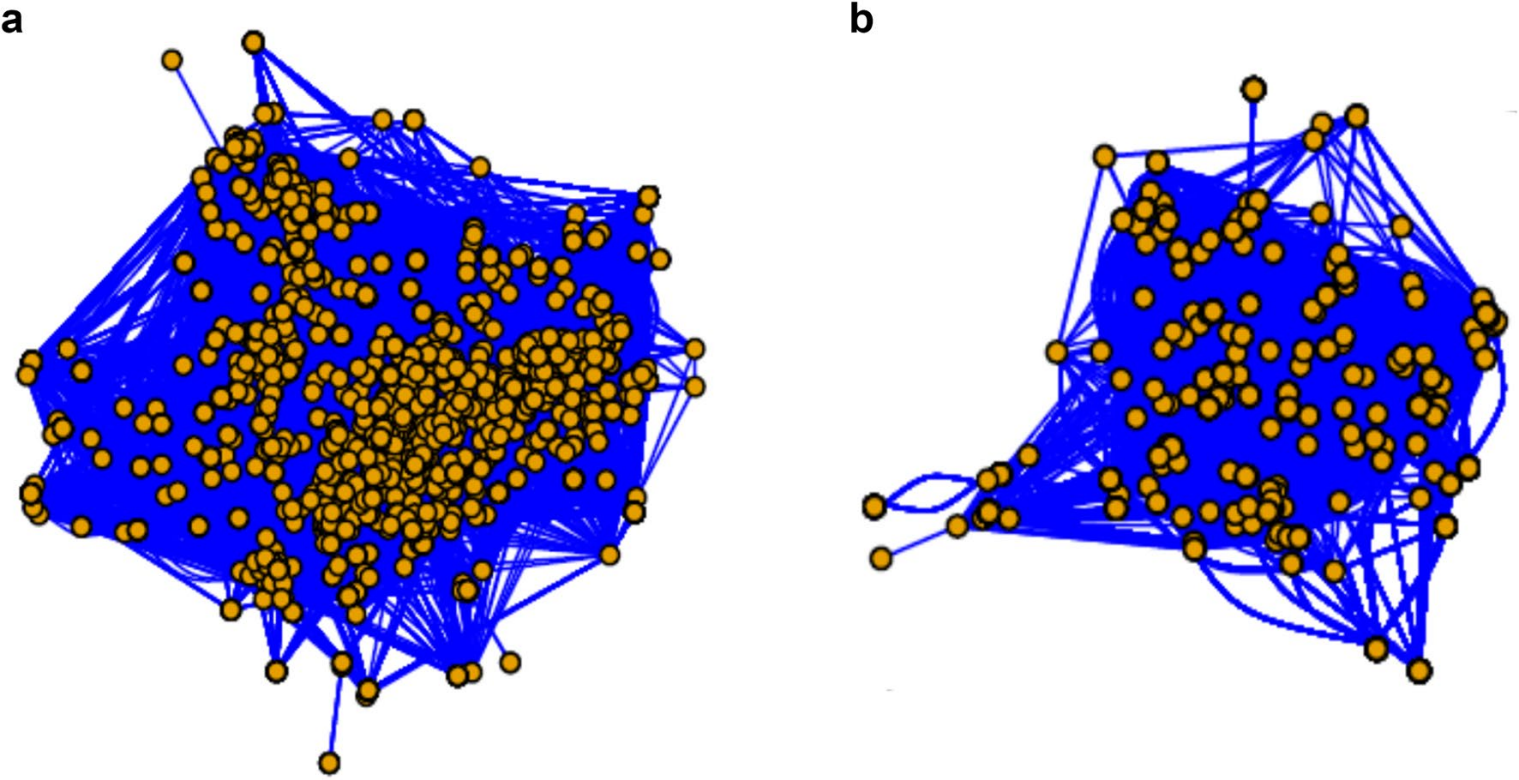
Graphical representation of social networks by the association between individuals of Audouin’s gulls in breeding patches comparing (**a**) the stability phase (2002–2011) and (**b**) the transition phase to colony collapse (2012–2017) (see Fig. 1). Each node represents an individual and each edge links those individuals that have bred in the same patch. We used the half-weight association index (HWI) to estimate the strength of the relationship between pairs of individuals. The HWI is more suitable than other metrics when not all individuals within each group have been identified^82,83^.

Accordingly, when assessing the social ties with dependent regression terms in the AME function, we showed that the probability of breeding together during the second sub-period (2007–2011) depended on whether they have bred previously together in the first sub-period (2002–2006), with a statistically significant coefficient of regression parameter (Table 1; Fig. 2). When analysing this data set with permutated data, we concluded that there was indeed a non-random association of individuals within the patches, with our statistic being among the 5% extreme values.

**Table 1:** Results of the AME regression function to test if there were social ties between individuals while breeding during the stable period and during the transition phase to the collapse period.

	Stable period				Transition to collapse period			
	pmean	psd	z-stat	P value	pmean	psd	z-stat	P value
intercept	− 1.467	0.087	16.808	< 0.001	− 0.231	0.018	− 12.629	0.000
.dyad	6.420	0.328	19.576	< 0.001	− 0.004	0.022	− 0.188	0.851

The null hypothesis is that individuals aggregate annually at random for breeding. “.dyad”: coefficient of the dependent regression term considering the previous dyadic relationship between individuals; “pmean”: posterior mean estimate; “psd”: posterior standard deviation; “z-stat “: nominal z-score.

### Impact of perturbations on social cohesion

When we analysed the social ties during the transition to collapse phase, we observed that the probability of breeding together during the period 2012–2017 did not depend on whether they have bred in the same patch the five previous years (χ_1_^2^ = 1.8143, P value = 0.178) (Supplementary Table S1) and we could not reject the hypothesis of a random association between individuals during that period. Besides, the SNA approach showed that breeding aggregations in Audouin’s gull during the transition phase period did not depend on whether they have bred in the same patch the five previous years (Table 1; Fig. 2).

## Discussion

By studying a particular ecological system of a colonial long-lived species that experiences a perturbation regime, we showed that individuals do not associate at random but there are social ties among individuals that persist over the years when conditions are unperturbed. Additionally, we observed that perturbations may decrease social cohesion in animal populations.

The characteristic breeding behaviour of the study species that aggregate in patches that change annually, allowed us to show that there is some group stability, with individuals establishing social ties that persist over time. Even if we cannot rule out that habitat preference is behind the observed individual non-random aggregations, we consider this hypothesis improbable in our study system as there is no obvious habitat differences between the patches. Our study system resembles what was recorded for Slender-billed gulls (*Chroicocephalus genei*), a colonial breeder with weak inter-annual breeding-site fidelity: some individuals bred in the same patch across breeding seasons and some social groups showed tenacity despite the colony often moving every year^46^. Group stability can emerge as a product of network self‐organization, although may then provide the necessary conditions for the evolution of other social processes^47,48^. Our results would support the idea that social aggregation during breeding would provide other advantages than the mere defence against predators^49,50^, such as social information sharing (e.g.^51–53^), especially in colonial seabirds that evolved in changing and unpredictable environments^42^. Social information sharing is also crucial for decision-making in risky behaviours, such as dispersal, and previous studies showed that the perturbed regime in our study site caused dispersal to other sites, including the colonization of new habitats^41,54^. In our case study, sociality may have played a major role in driving dispersal and thus population dynamics, both during the exponential growth after colonization and the collapse after the perturbation regime^24,42^. This idea is also reinforced with a mechanistic dynamical model that shows that population dynamics of Audouin’s gulls at the study site can only be explained by dispersal runaway caused by social copying^24^.

The importance of social information compared to private information increases under perturbations, even when the quality of social information does not increase compared to a non-perturbed regime^55,56^. For instance, Maldonado et al.^57^ show that experimental disturbances applied to a social bird may impact its foraging efficiency by social instability caused by the split of social groups. In colonial birds, breeding failure, which is a proxy of environmental stress, may trigger splitting of the social groups (e.g.^46^). At the demographic level, the alteration of social network structure may affect the behaviour of populations. For instance, under stress conditions, sociality may operate through feedback loops such as social copying for dispersal, causing non-linear population dynamics and playing a critical role in the resilience of populations (e.g.^24^). We showed here that after a perturbation, not only the number of individuals in the population may decrease (by increased mortality or dispersal) but also its social cohesion, likely reducing but also altering the information transfer within the social network composed by those individuals that remain in the site where perturbation occurs. Among other demographic processes, dispersal may alter social connections of both individuals remaining and those dispersing, with consequences for social network structure^45^. The perturbation regime suffered by the study population has likely triggered a social transition^58^ in collective behaviour from philopatric to dispersal and with the fast diffusion of innovations such as the colonization of harbours by a large number of individuals, a habitat safe from predators never occupied before^54^. Previous studies have shown that responses of populations to perturbations may also depend on individual personalities in the population^22,59–61^. For example, dispersers are different from non-dispersing individuals for a suite of phenotypic traits, including their behavioural profile^62–64^. Heterogeneities in personalities for dispersal decision-making may have also played a role in our studied population, with most individuals dispersing to other sites after a period of disturbance, while some individuals remaining philopatric. This change may have also further consequences for social network stability, as performance in social groups may improve with heterogeneity in individual personalities^64,65^.

Our study opens new research avenues about the resilience of social organisms under perturbations; if perturbations affect social cohesion and heterogeneity in personalities in the population, we may wonder whether this population would be equally resilient to future perturbations. Additionally, in our study population, sociality seemed to operate not right after the first perturbation episode but after cumulative maintained perturbation^54^; would the type of perturbation, either pulse or in regime^66^ influence the response of social groups? Further studies considering individual heterogeneity, sociality, and different types of perturbations should be carried out to improve our understanding on the demographic consequences of the breaking down of social ties for population dynamics and resilience in social species.

## Material and methods

### Study species and study area

The Audouin’s gull is a long-lived seabird with more than 80% of the global population breeding in the western Mediterranean (https://www.iucnredlist.org/details/22694313/0^43^. The species was critically endangered until the early 1980s when it colonized a new site, the Punta de la Banya in the Ebro Delta (Fig. 2). Here, the large availability of both suitable breeding habitat and food resulted in rapid and exponential growth, ending with the site holding more than 70% of the total world population in 2006. The global population dynamics were mainly driven by this colony and after the exponential growth, the species was downgraded to a conservation category of “least concern”^67^. However, the Punta de la Banya colony is now collapsing and even if the species is colonizing new sites, the global population is decreasing at a 5% annual rate^43^. In 1997, first carnivores (mainly foxes, but also badgers, beech martens, and least weasels) arrived at Punta de la Banya, likely due to their increasing densities in recent decades^68^ and the attractiveness of the site in terms of food availability and lack of competition. Since then the site has been perturbed by the presence of carnivores.

**Figure 3:**
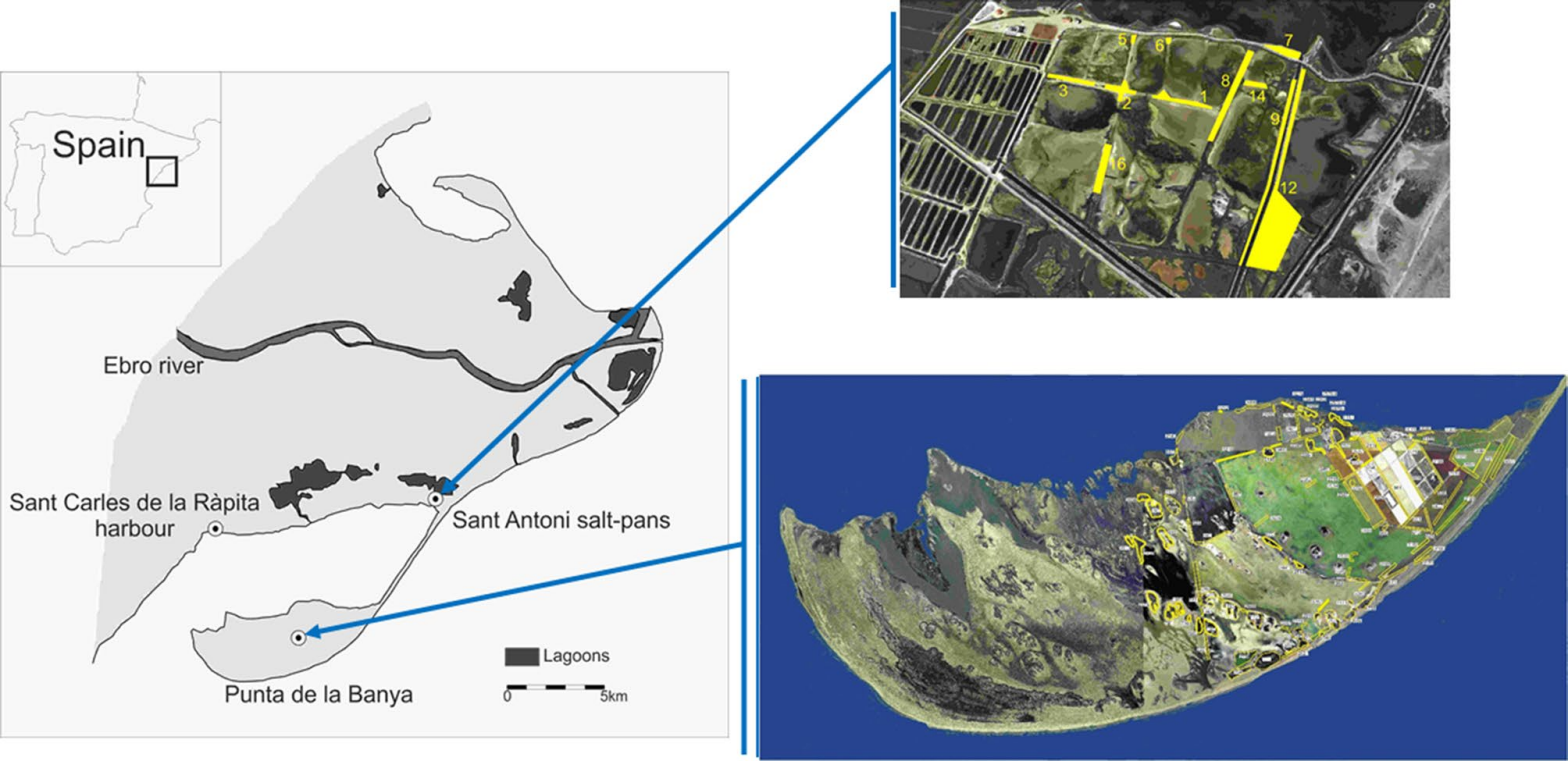
Map of the study area comprising the 3 main colonies and the distribution of patches within colonies during the study period at (**a**) Sant Antoni and (**b**) Punta de la Banya. Sant Carles de la Rápita colony is considered to have only one patch. Map made by the authors from the Orthophoto of Catalonia 1:5000 of the Institut Cartogràfic i Geològic de Catalunya (ICGC)( https://www.icc.cat/vissir3/), used under a CC BY 4.0 license with the use of CorelDraw X5 v.15.2.0.686.

Annual censuses of breeding pairs at every patch within colonies at the Ebro Delta area have been carried out since colonization in 1981 to 2017 (Fig. 1, 3; Supplementary Table S2). In the Ebro Delta there are three colonies: Punta de la Banya colonized in 1981 and occupied throughout the study period, Sant Carles de la Ràpita harbour, occupied from 2011 to 2015, and Sant Antoni, occupied from 2013 up to now (Fig. 3; Supplementary Figure S1). Within colonies, individuals are distributed in patches^69^. As patch location may change from 1 year to another, we annually geolocalized, mapped and defined the breeding patches (Fig. 3; Supplementary Figure S2).

The species is monogamous, there is assortative mating by age, and from an evolutionary point of view is a bet-hedger, laying commonly 3 eggs, although few chicks survive, except in years with high food availability, when the strength of density-dependence is low^70^.

### Individual data

During 1988–2017 a total of 30,290 individuals were captured and ringed as chicks at the Punta de la Banya^71,72^. From 2002 to 2017, resightings were made using spotting scopes from a distance all over the western Mediterranean with a total of 63,106 resights in the study area and 5593 different individuals resighted. Each year we recorded the breeding patch for each individual. To make sure that individuals were breeding and that they did so in a particular patch, we only selected those individuals seen during the breeding season in a particular patch showing unequivocal breeding behaviour: specifically, individuals making alarm calls, incubating eggs, or with chicks. Even if monitoring of the species started in 1988, resights made before 2002 could not be included in this analysis because we did not record breeding behaviour that assures us that the individual was breeding on that particular patch or just resting or prospecting there. After the selective filter of unequivocal breeders, our final database included 3548 individuals.

We considered the breeding association between two individuals when individuals were recorded breeding in the same patch the same year, and not associated if they were breeding in different patches. If one of the individuals was missed or individuals do not occur in overlapping timeframes, were considered as missing data.

### SNA framework

Our social network, defined as the observed pattern of breeding association, was constructed taking individuals (N = 3548) as the nodes of the network and each edge dyad (i.e. two individuals) representing the fact that individuals breed in the same patch. We ended with a global sociomatrix, i.e. the matrix representation of the dyadic relationships among individuals, of 3548* 3548. Edges showed if two individuals bred in the same patch at least once in a certain period (see below). The network was not directional. Based on previous results on population dynamics, and the population size of this species and colony^43,54^, we divided our dataset into two main periods: a period defined as “stable phase” from 2002 to 2011, and a period of “transition phase to collapse”, from 2012 to 2017 (Fig. 1).

We used the recently developed AME function from the AMEN package^73,74^, which can be applied to binary, ordinal, and continuous network data. This new approach is a random-effects regression model; uses an iterative Markov chain Monte Carlo (MCMC) algorithm that provides Bayesian inference of the parameters in the social relations regression model (SRM^75^) using additive and multiplicative effects and combining the linear regression model with the covariance structure of the SRM^74^. The AME method, not currently used in research on animal social networks is also able to cope with missing and censored data, our data set complying with the assumption that individuals are missing at random. Through Markov chain Monte Carlo algorithm, the AME function approximates the joint posterior distribution of all unknown quantities in the system, including parameters and missing data. Specifically, as in almost all Bayesian modeling methods, at each iteration of the Markov chain, values for missing data are iteratively simulated from their conditional distribution, given the observed data values and the current values of the unknown model parameters. For that being accurate, “individuals should be missing at random”, an assumption we feel confident with because we have no reason to suspect that those individuals that we fail to observe each year in the field are not a random sample of all the individuals. Coping with missing data is highly relevant when analysing sociality on wild populations, as detection rate for individuals is almost always imperfect, and properly controlling for missed observations is a very important step in social network analysis^76,77^. To create and visualize our networks we used the packages Amen^73^, Asnipe^78^, gdata^79^ and igraph in R^80^.

### Occurrence of social ties that persist over time

We investigated if individuals create social ties that persist over time longer than one breeding occasion by means of two approaches: (1) contingency tables and (2) the inclusion of time-dependent regression terms in the AME modelling framework^73^ (see the previous section). We used both methods because this is the first application of the AME approach in an ecological context. We analysed data of the period of stability, from 2002 to 2011, dividing this period into two sub-periods of 5 years (2002–2006 and 2007–2011). In the contingency table approach, we tested if the probability of two individuals breeding in the same patch at least once during a 5-year period (2007–2011) was independent of having bred in the same patch at least once in the previous 5-year period (2002–2006). We built a 3 × 3 table of frequencies, showing the frequencies of two individuals breeding or not together at least once during the second sub-period depending on whether they bred in the same patch or not at least once during the first sub-period, pulling apart those dyads with missing data. We then tested for deviation of random frequencies by ${\chi }^{2}$ test.

With the SNA approach, we analysed the social ties between individuals using the AME function provided in the Amen R-package and including data from the first period (five previous years) as predictors of the association during the second period. We considered that this time window was not too large to include important death events, but large enough to account for the imperfect detection of individuals. To achieve convergence, we increased the number of iterations to 100,000 from the default value of 10,000 and lengthened the burn-in period to 500.

### Impact of perturbations on social cohesion

To assess if perturbations affected social structure in this species, we analysed as previously, with both the contingency table approach and the SNA approach, the social ties during the “transition phase to collapse” (2012–2017). To do so, we tested if the probability of breeding in the same patch in this phase (2012–2017) was independent of having bred in the same patch during five previous years (2007–2011). We then compared these results from those previously observed during the “stability phase”.

A potential concern was the reduced power during the collapse period because the number of individuals decreased from the stability period. In order to have similar power in both analyses, we performed the contingency table analysis during the stability period by drawing at random a number of observed associations equal to the number of observed associations during the collapse. We did this repeatedly (1000 times) and calculated which percentage of times the resulting chi-square was significant at the 5% level.

Regarding the SNA approach, it is advised^81^ to do permutations on the raw data prior to the analysis and compare the result of some relevant statistic obtained with the original data to the distribution of the same statistic over the permutations. We chose the regression coefficient of the association of a dyad on the previous association of the same dyad as our statistic of social cohesion. This statistic is provided by the function ame of the package amen^73^. We calculated this statistic on the original data. Then, within each year, we rearranged randomly the individuals among the patches, keeping the same number of individuals within each patch. We did this 200 times and calculated each time the regression coefficient. Then, we situated the value of the regression coefficient from the original data among the distribution of regression coefficients from the permutated data and examined how extreme it was. If it was among the 5% extreme values, we concluded that there was indeed a non-random association of individuals within the patches.

## Supplementary information

Supplementary Information

## Data Availability

Data is available via CSIC repository at https://hdl.handle.net/10261/216539.

## References

[1] Holling CS (1973). Resilience and stability of ecological systems. Annu. Rev. Ecol. Syst..

[2] Dai L, Korolev KS, Gore J (2015). Relation between stability and resilience determines the performance of early warning signals under different environmental drivers. Proc. Natl. Acad. Sci..

[3] Dakos V, Carpenter SR, van Nes EH, Scheffer M (2014). Resilience indicators: Prospects and limitations for early warnings of regime shifts. Philos. Trans. R. Soc. B Biol. Sci..

[4] Colchero F (2018). The diversity of population responses to environmental change. Ecol. Lett..

[5] Coulson T (2017). Data from: Modeling adaptive and nonadaptive responses of populations to environmental change. Am. Nat..

[6] Donohue I (2016). Navigating the complexity of ecological stability. Ecol. Lett..

[7] Fernández-Chacón A (2013). When to stay, when to disperse and where to go: Survival and dispersal patterns in a spatially structured seabird population. Ecography.

[8] Sterk M, van de Leemput IA, Peeters ET (2017). How to conceptualize and operationalize resilience in socio-ecological systems?. Curr. Opin. Environ. Sustain..

[9] Brand FS, Jax K (2007). Focusing the meaning(s) of resilience: Resilience as a descriptive concept and a boundary object. Ecol. Soc..

[10] Barrett L, Henzi SP, Lusseau D (2012). Taking sociality seriously: The structure of multi-dimensional social networks as a source of information for individuals. Philos. Trans. R. Soc. B Biol. Sci..

[11] Centola, D. *How Behavior Spreads: The Science of Complex Contagions*. (2018).

[12] Firth JA (2020). Considering complexity: Animal social networks and behavioural contagions. Trends Ecol. Evol..

[13] Kerth G, Perony N, Schweitzer F (2011). Bats are able to maintain long-term social relationships despite the high fission–fusion dynamics of their groups. Proc. R. Soc. B Biol. Sci..

[14] Rosenthal SB, Twomey CR, Hartnett AT, Wu HS, Couzin ID (2015). Revealing the hidden networks of interaction in mobile animal groups allows prediction of complex behavioral contagion. Proc. Natl. Acad. Sci..

[15] Snijders L, Blumstein DT, Stanley CR, Franks DW (2017). Animal social network theory can help wildlife conservation. Trends Ecol. Evol..

[16] Webber QMR, Vander Wal E (2018). An evolutionary framework outlining the integration of individual social and spatial ecology. J. Anim. Ecol..

[17] Sueur C, Mery F (2017). Social Interaction in Animals: Linking Experimental Approach and Social Network Analysis.

[18] LaBarge LR, Allan ATL, Berman CM, Margulis SW, Hill RA (2020). Reactive and pre-emptive spatial cohesion in a social primate. Anim. Behav..

[19] Firth JA (2017). Wild birds respond to flockmate loss by increasing their social network associations to others. Proc. R. Soc. B Biol. Sci..

[20] Farine DR (2019). Structural trade-offs can predict rewiring in shrinking social networks. J. Anim. Ecol..

[21] Maldonado-Chaparro AA, Alarcón-Nieto G, Klarevas-Irby JA, Farine DR (2018). Experimental disturbances reveal group-level costs of social instability. Proc. R. Soc. B Biol. Sci..

[22] Puga-Gonzalez I, Sosa S, Sueur C (2019). Social style and resilience of macaques’ networks, a theoretical investigation. Primates.

[23] Williams R, Lusseau D (2006). A killer whale social network is vulnerable to targeted removals. Biol. Lett..

[24] Oro D (2020). Perturbation, Social Feedbacks, and Population Dynamics in Social Animals.

[25] Firth JA, Sheldon BC (2015). Experimental manipulation of avian social structure reveals segregation is carried over across contexts. Proc. R. Soc. B Biol. Sci..

[26] Genton C (2015). How Ebola impacts social dynamics in gorillas: A multistate modelling approach. J. Anim. Ecol..

[27] Leu ST, Farine DR, Wey TW, Sih A, Bull CM (2016). Environment modulates population social structure: Experimental evidence from replicated social networks of wild lizards. Anim. Behav..

[28] Silk J, Cheney D, Seyfarth R (2013). A practical guide to the study of social relationships: A practical guide to the study of social relationships. Evol. Anthropol. Issues News Rev..

[29] Brown CR (2016). The ecology and evolution of colony-size variation. Behav. Ecol. Sociobiol..

[30] Rolland C, Danchin E, de Fraipont M (1998). The evolution of coloniality in birds in relation to food, habitat, predation, and life-history traits: A comparative analysis. Am. Nat..

[31] Shizuka D (2014). Across-year social stability shapes network structure in wintering migrant sparrows. Ecol. Lett..

[32] Brandl HB, Griffith SC, Farine DR, Schuett W (2019). Wild zebra finches that nest synchronously have long-term stable social ties. J. Anim. Ecol..

[33] Moreno, J. L. *Who Shall Survive?: A New Approach to the Problem of Human Interrelations* (Nervous and Mental Disease Publishing Co, New York, 1934). .

[34] Scott J (1988). Social network analysis. Sociology.

[35] Croft DP, James R, Krause J (2008). Exploring Animal Social Networks.

[36] Farine DR, Whitehead H (2015). Constructing, conducting and interpreting animal social network analysis. J. Anim. Ecol..

[37] Ward A, Webster M (2016). Sociality: The Behaviour of Group-Living Animals.

[38] Whitehead, H. *Analyzing Animal Societies Quantitative Methods for Vertebrate Social Analysis*. (2014).

[39] James R, Croft DP, Krause J (2009). Potential banana skins in animal social network analysis. Behav. Ecol. Sociobiol..

[40] Hasenjager MJ, Dugatkin LA, Naguib M (2015). Chapter three—social network analysis in behavioral ecology. Advances in the Study of Behavior.

[41] Payo-Payo A (2018). Predator arrival elicits differential dispersal, change in age structure and reproductive performance in a prey population. Sci. Rep..

[42] Martínez-Abraín A, Oro D, Forero MG, Conesa D (2003). Modeling temporal and spatial colony-site dynamics in a long-lived seabird. Popul. Ecol..

[43] Genovart M, Oro D, Tenan S (2018). Immature survival, fertility, and density dependence drive global population dynamics in a long-lived species. Ecology.

[44] Almaraz P, Oro D (2011). Size-mediated non-trophic interactions and stochastic predation drive assembly and dynamics in a seabird community. Ecology.

[45] Shizuka D, Johnson AE (2019). How demographic processes shape animal social networks. Behav. Ecol..

[46] Francesiaz C (2017). Familiarity drives social philopatry in an obligate colonial breeder with weak interannual breeding-site fidelity. Anim. Behav..

[47] Cantor M, Farine DR (2018). Simple foraging rules in competitive environments can generate socially structured populations. Ecol. Evol..

[48] Cantor, M. *et al. Animal social networks: Revealing the causes and implications of social structure in ecology and evolution*. https://osf.io/m62gb (2019). 10.32942/osf.io/m62gb.

[49] Anderson DJ, Hodum PJ (1993). Predator behavior favors clumped nesting in an oceanic seabird. Ecology.

[50] Oro D (1996). Colonial seabird nesting in dense and small sub-colonies: An advantage against aerial predation. Condor.

[51] Gil MA, Hein AM, Spiegel O, Baskett ML, Sih A (2018). Social information links individual behavior to population and community dynamics. Trends Ecol. Evol..

[52] Lewanzik D, Sundaramurthy AK, Goerlitz HR (2019). Insectivorous bats integrate social information about species identity, conspecific activity and prey abundance to estimate cost–benefit ratio of interactions. J. Anim. Ecol..

[53] Doligez B (2002). Public information and breeding habitat selection in a wild bird population. Science.

[54] Payo-Payo A (2017). Colonisation in social species: The importance of breeding experience for dispersal in overcoming information barriers. Sci. Rep..

[55] Arganda S, Pérez-Escudero A, de Polavieja GG (2012). A common rule for decision making in animal collectives across species. Proc. Natl. Acad. Sci..

[56] Pérez-Escudero A, de Polavieja GG (2017). Adversity magnifies the importance of social information in decision-making. J. R. Soc. Interface.

[57] Maldonado-Chaparro AA, Blumstein DT, Armitage KB, Childs DZ (2018). Transient LTRE analysis reveals the demographic and trait-mediated processes that buffer population growth. Ecol. Lett..

[58] Pruitt JN (2018). Social tipping points in animal societies. Proc. R. Soc. B.

[59] Dall SRX, Houston AI, McNamara JM (2004). The behavioural ecology of personality: Consistent individual differences from an adaptive perspective. Ecol. Lett..

[60] Doering GN, Scharf I, Moeller HV, Pruitt JN (2018). Social tipping points in animal societies in response to heat stress. Nat. Ecol. Evol..

[61] Wolf M, van Doorn GS, Leimar O, Weissing FJ (2007). Life-history trade-offs favour the evolution of animal personalities. Nature.

[62] Clobert J, Le Galliard J-F, Cote J, Meylan S, Massot M (2009). Informed dispersal, heterogeneity in animal dispersal syndromes and the dynamics of spatially structured populations. Ecol. Lett..

[63] Cote J, Clobert J, Brodin T, Fogarty S, Sih A (2010). Personality-dependent dispersal: Characterization, ontogeny and consequences for spatially structured populations. Philos. Trans. R. Soc. B Biol. Sci..

[64] Fogarty S, Cote J, Sih A (2011). Social personality polymorphism and the spread of invasive species: A model. Am. Nat..

[65] O’Shea-Wheller TA, Masuda N, Sendova-Franks AB, Franks NR (2017). Variability in individual assessment behaviour and its implications for collective decision-making. Proc. R. Soc. B Biol. Sci..

[66] Nimmo DG, Mac Nally R, Cunningham SC, Haslem A, Bennett AF (2015). Vive la résistance: Reviving resistance for 21st century conservation. Trends Ecol. Evol..

[67] IUCN. Larus audouinii: BirdLife International: The IUCN Red List of Threatened Species 2018: e.T22694313A132541241. (2018). 10.2305/IUCN.UK.2018-2.RLTS.T22694313A132541241.en.

[68] Martínez-Abraín A, Jiménez J, Oro D (2019). Pax Romana: ‘refuge abandonment’ and spread of fearless behavior in a reconciling world. Anim. Conserv..

[69] Genovart M, Jover L, Ruiz X, Oro D (2003). Offspring sex ratios in subcolonies of Audouin’s gull, Larus audouinii, with differential breeding performance. Can. J. Zool..

[70] Oro D, Ogilvie MA (1998). Audouin’s gull account. The Birds of Western Palearctic.

[71] Genovart M, Pradel R, Oro D (2012). Exploiting uncertain ecological fieldwork data with multi-event capture-recapture modelling: An example with bird sex assignment. J. Anim. Ecol..

[72] Oro D, Tavecchia G, Genovart M (2010). Comparing demographic parameters for philopatric and immigrant individuals in a long-lived bird adapted to unstable habitats. Oecologia.

[73] Hoff, P. D. Additive and multiplicative effects network models. arXiv:180708038 Stat (2018).

[74] Minhas, S., Hoff, P. D. & Ward, M. D. Inferential approaches for network analyses: AMEN for latent factor models. arXiv:161100460 Stat (2016).

[75] Warner RM, Kenny DA, Stoto M (1979). A new round robin analysis of variance for social interaction data. J. Pers. Soc. Psychol..

[76] Gimenez O (2019). Inferring animal social networks with imperfect detection. Ecol. Model..

[77] Hoppitt WJE, Farine DR (2018). Association indices for quantifying social relationships: How to deal with missing observations of individuals or groups. Anim. Behav..

[78] Farine DR (2013). Animal social network inference and permutations for ecologists in R using asnipe. Methods Ecol. Evol..

[79] Warnes,GR, Bolker, G, Gorjanc, G & Grothendieck, G. gdata: Various R programming tools for data manipulation. *R package version* (2014).

[80] Csardi G, Nepusz T (2006). The igraph software package for complex network research. InterJournal.

[81] Farine DR (2017). A guide to null models for animal social network analysis. Methods Ecol. Evol..

[82] Ginsberg JR, Young TP (1992). Measuring association between individuals or groups in behavioural studies. Anim. Behav..

[83] Cairns SJ, Schwager SJ (1987). A comparison of association indices. Anim. Behav..

