# Supplementary material for: Decrease in social cohesion in a colonial seabird under a perturbation regime
Source: Sci Rep. 2020 Oct 30;10:18720. doi: 10.1038/s41598-020-75259-3 (PMC7603481; doi:10.1038/s41598-020-75259-3)
Supplement: Supplementary file 1 — Supplementary Information [file 41598_2020_75259_MOESM1_ESM.pdf]

Supplementary Information

Decrease in social cohesion in a colonial seabird under a perturbation regime

Genovart, M. [a,b] *, Gimenez, O. c, Bertolero, A.[d], Choquet, R. [c], Oro, D. [a,b], Pradel, R. [c]

[a] CEAB (CSIC), Accés Cala Sant Francesc 14, 17300 Blanes, Spain

[b] IMEDEA (CSIC-UIB), Miquel Marquès 21, 07190 Esporles, Spain

[c] CEFE, CNRS, Univ. Montpellier, Univ. Paul Valéry Montpellier 3, EPHE, IRD, 34293 Montpellier, France

[d] Associació Ornitològica Picampall de les Terres de l'Ebre, 43580 Deltebre, Spain

* Corresponding author: m.genovart@csic.es

Figure S1. Annual number of breeding pairs at the three colonies of the Ebro Delta since colonization of the area in 1981.

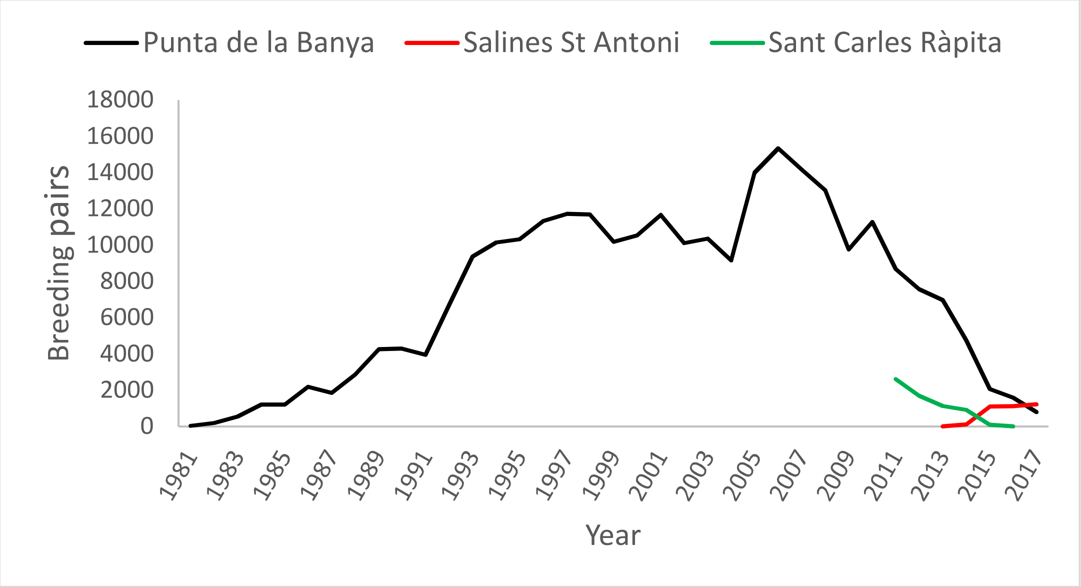

Figure S2. Example of annual patch aggregations at Punta de la Banya colony during a) the first year of the study period (2002) and b) the last year (2017).

a)

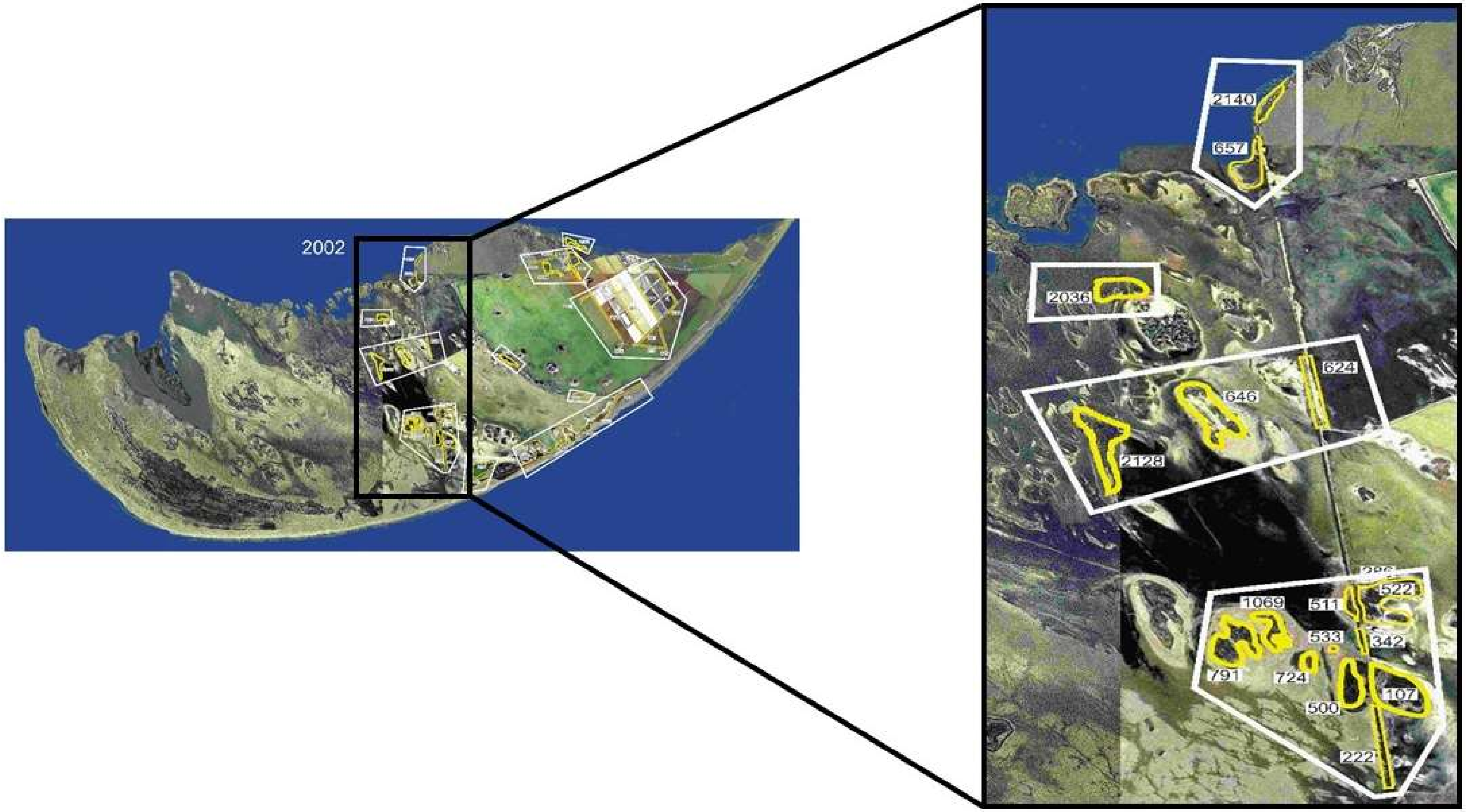

b)

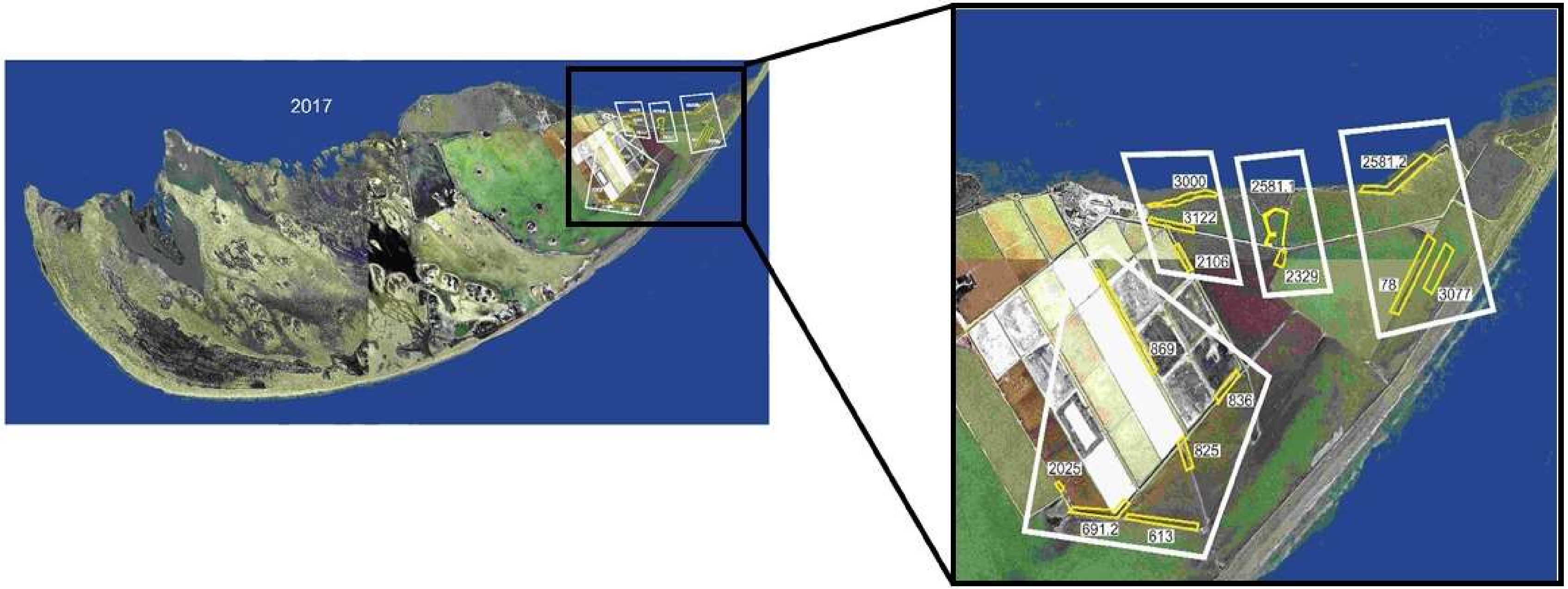
2017
3000
3122
2106
2581.1
2329
2581.2
78
3077
869
836
825
2025
691.2
613

Table S1. Contingency tables for testing if breeding aggregation in patches is done at random. Tables show the frequencies of two individuals breeding or not together at least once during a 5-year period depending on whether they bred together or not at least once during the previous 5-year period, and pulling apart those dyads with missing data. Table a) shows the frequencies during the Stability period (2002-2011), and we found that individuals that bred in the same patch during the sub-period 2002-2006 had a higher probability of breeding together during the sub-period 2007-2011 (χ^2= 64.685, $P < 0.0001$). When randomly reducing sample size of the data set, results were still statistically significant in more than 95% of the cases (1000 randomizations). Table b) shows the frequencies during the Period of crisis (2012-2017). When we analysed the social ties during the transition to collapse phase, we observed that the probability of breeding together during the period 2012-2017 did not depend on whether they have bred in the same patch the five previous years (χ2 =1.814, p-value = 0.178)

a)

**Stability period**	Do not breed together in the period 2007-2011	Breed together in the period 2007-2011	Missing data of at least one member during 2007-2011	Total
Had never bred together in the period 2002-2006	6173	1239	414161	421573
Had bred together in the period 2002-2006	2452	748	149075	152275
Missing data of at least one member during 2002-2006	179459	40899	5494627	5714985
	188084	42886	6057863	6288833

b)

**Crisis period**	Do not breed together in the period 2012-2017	Breed together in the period 2012-2017	Missing data of at least one member during 2012-2017	Total
Had never bred together in the period 2007-2011	1325	684	186520	188529
Had bred together in the period 2007-2011	324	193	41924	42441
Missing data of at least one member during 2007-2011	125133	56412	2167343	2348888
	126782	57289	2395787	2579858

Table S2. Description of annual patch aggregations at Punta de la Banya colony during the study period (2002-2017). Mean N: Mean number of breeding pairs in a patch; SD: standard deviation of the number of breeding pairs in each patches in a year; total N: total number of breeding pairs in the colony; Patches: Number of patches.

	**2002**	**2003**	**2004**	**2005**	**2006**	**2007**	**2008**	**2009**	**2010**	**2011**	**2012**	**2013**	**2014**	**2015**	**2016**	**2017**	**2002-2017**
**Mean N**	919.91	873.17	705.23	1075.5	1184.3	852.94	723.94	574.24	663	627.61	545.53	676.92	364	407.5	339.25	253.38	**674.15**
**SD N**	1250.4	1023.9	1021.4	1116.9	1500.2	1070.6	928.12	639.81	816.64	793.46	556.39	552.28	389.09	515.45	242.14	277.15	**361.73**
**Patches**	11	12	13	13	13	17	18	17	17	18	17	12	16	8	8	8	**13.63**
**Total N**	10119	10478	9168	13981	15396	14500	13031	9762	11271	11297	9274	8123	5824	3260	2714	2027	**9389.06**
